# Supplementary material for: Modelling Skylarks (Alauda arvensis) to Predict Impacts of Changes in Land Management and Policy: Development and Testing of an Agent-Based Model
Source: PLoS One. 2013 Jun 6;8(6):e65803. doi: 10.1371/journal.pone.0065803 (PMC3675089; doi:10.1371/journal.pone.0065803)
Supplement: Supporting Information S4 — The skylark ODdox as a zipped archive. (ZIP) [file pone.0065803.s004.zip › Skylark_ODdox/functions_0x69.html]

ALMaSS Skylark ODdox: Class Members


ALMaSS Skylark ODdox
 2.0


- Main Page
- Related Pages
- Classes
- Files

- Class List
- Class Index
- Class Hierarchy
- Class Members

- All
- Functions
- Variables
- Typedefs

- a
- b
- c
- d
- e
- f
- g
- h
- i
- j
- k
- l
- m
- n
- o
- p
- r
- s
- t
- u
- v
- w
- x
- y
- z
- ~

Here is a list of all class members with links to the classes they belong to:

### - i -

- IDMap()
  : IDMap
- ImpactedProbe()
  : Population\_Manager
- ImpactProbeReport()
  : Population\_Manager
- incNoChickDeaths()
  : Skylark\_Population\_Manager
- incNoFledgeDeaths()
  : Skylark\_Population\_Manager
- incNoPestEffects()
  : Skylark\_Population\_Manager
- incTotalEggs()
  : Skylark\_Population\_Manager
- incTotalNestlings()
  : Skylark\_Population\_Manager
- incTotalPrefledgelings()
  : Skylark\_Population\_Manager
- IncTreatCounter()
  : Landscape
- index
  : tpct
- IndexArrayX
  : Population\_Manager
- Init()
  : Skylark\_Population\_Manager
  , MovementMap
  , MovementMap16
  , PositionMap
- InitiateManagement()
  : FarmManager
  , Farm
  , UserDefinedFarm
  , UserDefinedFarm36
- InitiateRodenticidePolyData()
  : RodenticideManager
- Insecticide()
  : LE
  , VegElement
- InsecticideTreat()
  : Farm
- InsectMortality()
  : LE
  , VegElement
- Insert()
  : SkQualGrid
- InSquare()
  : Skylark\_Base
- InvIntPartition()
  : UserDefinedFarm
- IsBadWeather()
  : Skylark\_Population\_Manager
- IsExtGridPositionValid()
  : SkTerritories
- IsGridPositionValid()
  : SkTerritories
- IsLast()
  : Population\_Manager
- IsRecentlyMown()
  : LE
- IsRecentlySprayed()
  : LE
- IsStockFarmer()
  : Farm
- IsValid()
  : SkTerritories


- Generated on Thu Jan 10 2013 13:15:39 for ALMaSS Skylark ODdox by
   1.8.1.1
